# The Sulfur Metabolite Lanthionine: Evidence for a Role as a Novel Uremic Toxin

**DOI:** 10.3390/toxins9010026

**Published:** 2017-01-10

**Authors:** Alessandra F. Perna, Miriam Zacchia, Francesco Trepiccione, Diego Ingrosso

**Affiliations:** 1 First Division of Nephrology, Department of Cardio-thoracic and Respiratory Sciences, University of Campania “Luigi Vanvitelli”, School of Medicine, via Pansini 5, Bldg 17, Naples 80131, Italy; miriam.zacchia@unina2.it (M.Z.); francesco.trepiccione@ana.au.dk (F.T.); 2 Department of Biochemistry, Biophysics and General Pathology, University of Campania “Luigi Vanvitelli”, School of Medicine, via Luigi de Crecchio 7, Naples 80138, Italy; diego.ingrosso@unina2.it

**Keywords:** lanthionine, homolanthionine, hydrogen sulfide, homocysteine, hemodialysis, uremic toxins

## Abstract

Lanthionine is a nonproteinogenic amino acid, composed of two alanine residues that are crosslinked on their β-carbon atoms by a thioether linkage. It is biosynthesized from the condensation of two cysteine molecules, while the related compound homolanthionine is formed from the condensation of two homocysteine molecules. The reactions can be carried out by either cystathionine-β-synthase (CBS) or cystathionine-γ-lyase (CSE) independently, in the alternate reactions of the transsulfuration pathway devoted to hydrogen sulfide biosynthesis. Low plasma total hydrogen sulfide levels, probably due to reduced CSE expression, are present in uremia, while homolanthionine and lanthionine accumulate in blood, the latter several fold. Uremic patients display a derangement of sulfur amino acid metabolism with a high prevalence of hyperhomocysteinemia. Uremia is associated with a high cardiovascular mortality, the causes of which are still not completely explained, but are related to uremic toxicity, due to the accumulation of retention products. Lanthionine inhibits hydrogen sulfide production in hepatoma cells, possibly through CBS inhibition, thus providing some basis for the biochemical mechanism, which may significantly contribute to alterations of metabolism sulfur compounds in these subjects (e.g., high homocysteine and low hydrogen sulfide). We therefore suggest that lanthionine is a novel uremic toxin.

## 1. Introduction

**Figure 1:**
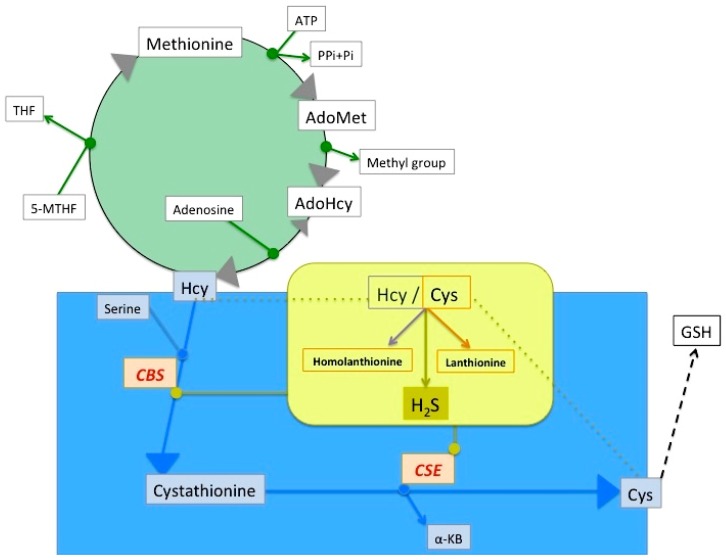
Sulfur amino acid metabolism, and its connections with the one carbon metabolism. The essential sulfur amino acid l-methionine is the precursor of homocysteine (Hcy) in the methionine-Hcy cycle (green), in which methionine is first enzymatically activated to *S*-adenosylmethionine (AdoMet) in an ATP-dependent reaction. AdoMet is the universal methyl donor for about 50 different methyltransferases. The AdoMet demethylated product, i.e., AdoHcy, is the immediate Hcy precursor in a fully reversible reaction. AdoHcy is a powerful competitive methyltransferase inhibitor, although under normal conditions, this inhibition is hampered by prompt removal of AdoHcy hydrolysis products (Hcy and adenosine). Hcy can be remethylated to methionine, in a reaction catalyzed by methionine synthase, a l-5-methyltetrahydrofolate-dependent (MTHF) enzyme, yielding tetrahydrofolate (THF). Hcy is also the precursor of l-cysteine through transsulfuration (blue), a two-step pyridoxal phosphate-dependent pathway, catalyzed by cystathionine-β-synthase (CBS) which condenses l-serine and Hcy, forming cystathionine, followed by its hydrolysis to l-cysteine (Cys) and α-keto-butyrate (α-KB), catalyzed by cystathionine-γ-lyase (CSE). Cys is in turn the precursor of glutathione (GSH). Both CBS and CSE may independently catalyze hydrogen sulfide (H_2_S; yellow) biosynthesis, by using Cys (or Hcy as an alternative substrate) (see also Figure 2). The partitioning of sulfur amino acid metabolism between transsulfuration and H_2_S formation is highly regulated. CBS and CSE are crucially regulated by many modulators, also including the availability of cystathionine produced by CBS. It has been reported that, under acute conditions, in human embryonic kidney cells, endoplasmic reticulum (ER) stress, induced by thapsigargin treatment, induces CSE and triggers a change from the transient induction of the complete transsulfuration pathway to preferential H_2_S production. An increase of CO, produced by heme-oxigenase-2 overexpression in these cells, inhibits CBS and, in the absence of ER stress, switches CSE activity from the cystathionine hydrolysis mode, where cystathionine is converted into cysteine (and eventually GSH), to H_2_S production from cysteine. The latter condition may mimic the consequences of chronic CBS-impairing mutations underpinning homocystinuria [9]. Conversely, in hemodialysis patients, both oxidative stress and cystathionine are increased [10].

Patients affected by chronic kidney disease, a growing population in the world, and especially patients on hemodialysis, display a high cardiovascular mortality, the causes of which are still elusive. Such a high cardiovascular mortality has been related to an increased prevalence of traditional risk factors and, also, to the presence of non-traditional risk factors which do not occur in the general population. Many of the non-traditional risk factors affecting high cardiovascular mortality in renal disease have been linked to uremic toxins or their derivatives. For example, sulfur amino acid metabolism (Figure 1) has been found altered in many ways in chronic kidney disease. One of the most classical findings, which is almost constantly associated with uremia, is hyperhomocysteinemia, the causative role of which has been debated, although the rationale for its inclusion in the list of uremic toxins was eventually influenced by its role as a cardiovascular risk factor and its effects on the vasculature [1]. Interesting, in this respect, is the China Stroke Primary Prevention Trial (CSPPT), which showed the benefits of folic acid, as a means to reduce homocysteinemia, in preventing stroke in Chinese adults with hypertension [2]. In addition, Xu et al. reported findings from a prespecified CSPPT substudy demonstrating that folic acid plus enalapril was more effective than enalapril alone in the secondary prevention of renal function decline in mild-to-moderate chronic kidney disease [3].

Moreover, a number of other compounds, strictly related to homocysteine, have been found elevated in renal disease patients and are now under scrutiny as potential uremic toxins, thus making the case for the involvement of multiple intermediates in the same metabolic pathways [4,5,6]. Various other compounds, in fact, which are metabolically related to methionine and one carbon metabolism, also represent putative or actual uremic toxins including, for example: *S*-adenosyl-l-homocysteine (AdoHcy; SAH), the in vivo l-homocysteine precursor and a powerful inhibitor of methyltransferases, and asymmetric dimethylarginine (ADMA, a methylated derivative of arginyl residues in proteins, which is released from protein breakdown). AdoHcy is particularly promising as a better predictor of cardiovascular toxicity than homocysteine itself ([7]; Figure 1). Alterations of folate, the one carbon-unit transporter, and of folate binding proteins have been reported to occur in uremia ([8]; Figure 1).

More recently, a derangement in the circulating levels of hydrogen sulfide (H_2_S), has been reported to take place in uremia (see below).

## 2. Lanthionine, A Side Product of H_2_S Biosynthesis and A Prospective Uremic Toxin

**Figure 2:**
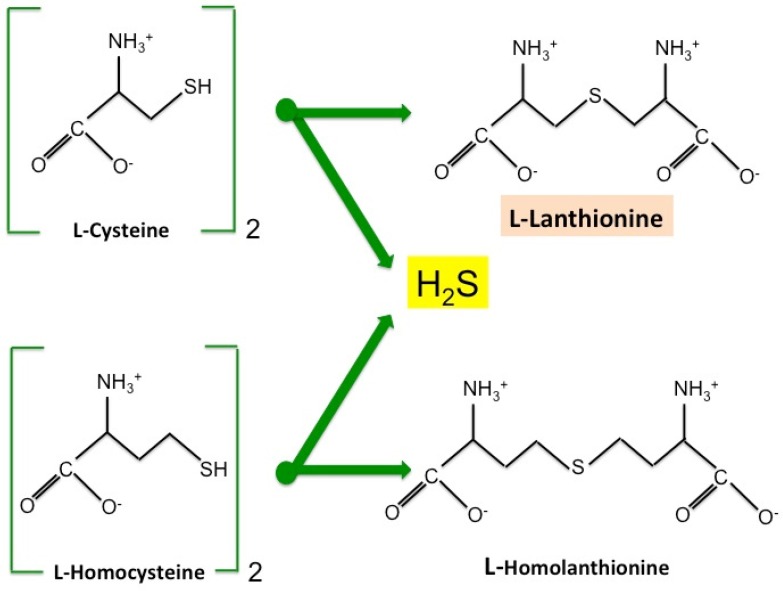
Biosynthesis of lanthionine and homolanthionine. l-Lanthionine is formed from the condensation of two cysteine molecules, while l-homolanthionine is formed from the condensation of two homocysteine molecules. While CBS and CSE act sequentially in the two-step transsulfuration pathway, either CBS or CSE can catalyze lanthionine or homolanthionine biosynthesis independently, under physiological conditions. However, cysteine is the prevalent substrate for H_2_S biosynthesis (yielding lanthionine) compared to homocysteine, although the contribution of the latter may increase under some conditions. This feature underscores the great versatility of CBS and CSE in regulating sulfur metabolism. It has been reported that carbon monoxide (CO)—a product of heme oxygenase 1 (HO-1)—may inhibit CBS under stress conditions (also possibly associated with homocystinuria due to CBS mutations or deletions), thus creating the conditions for a relative decrease of the complete transsulfuration pathway and an increase of H_2_S biosynthesis by CSE [9]. Conversely, we obtained evidence that in hemodialysis patients, lanthionine behaves as a retention product, despite a significant reduction of H_2_S in circulation [10].

We recently detected, in uremic patients undergoing hemodialysis, high concentrations of the sulfur amino acid derivative lanthionine, which increases by about two orders of magnitude with respect to normal subjects [10]. Lanthionine is a natural nonproteogenic amino acid, an analog of cysteine, consisting of two alanine residues crosslinked on their β-carbon atoms by a thioether linkage. The condensation of two molecules of cysteine (β-replacement reaction) produces H_2_S, an endogenous gas with important modulating properties, and lanthionine, while the condensation of two molecules of homocysteine produces H_2_S and homolanthionine (γ-replacement reaction) (Figure 2). The relevant enzymes are cystathionine-γ-lyase (CSE) and cystathione β-synthase (CBS), the main enzymes of the transsulfuration pathway leading to cysteine production and homocysteine detoxification, along with H_2_S production in alternate reactions, as previously mentioned [11,12,13,14]. Homolanthionine has also been found increased in uremia, although to a lesser extent compared with lanthionine [10]. Lanthionine and homolanthione have been therefore considered stable side-products of H_2_S metabolism. However, it can be argued, considering our findings in hemodialysis patients, that renal function is a very important variable to be taken into account. In patients on dialysis, these compounds can be considered, in fact, among the retention products, which are responsible in general for the uremic syndrome. H_2_S levels are instead lower in the blood of these patients, and this has been linked to a CSE down-regulation [15]. This is inscribed in the general derangement of sulfur metabolism present in uremic patients, encompassing high homocysteine and cysteine, low H_2_S, and high lanthionine and homolanthionine [16]. We also demonstrated that lanthionine can be effectively removed during hemodialysis, demonstrating that lanthionine is, for at least half of its concentration, in its free form [10].

## 3. Possible Causes of Increased Lanthionine

The high lanthionine levels in uremia could be ascribed to (1) increased production, or (2) reduced degradation or excretion. The substrates of H_2_S synthesis, homocysteine and cysteine, are indeed increased in uremic patients as mentioned, so that an increase in the biosynthesis of H_2_S could be theoretically hypothesized to occur. In addition, CBS can alternatively catalyze lanthionine synthesis through a β-substitution reaction, by condensation of cysteine and serine, but in this reaction a H_2_O molecule is produced instead of H_2_S [12,17]. Therefore, increased production of lanthionine and homolanthionine, independent from H_2_S generation, cannot be excluded to take place in uremia, in the face of reduced plasma H_2_S levels, because the above-mentioned reaction does not produce H_2_S. It is also possible that H_2_S is produced in excess and rapidly degraded. We previously showed a down-regulation of CSE in CKD, consistently associated with a decrease of blood sulphemoglobin, a marker of chronic exposure to H_2_S [15]; these data were mirrored by similar results in a CKD rat model [18]. Therefore, overproduction of lanthionine is not a very likely possibility in uremia. Rather, our data show that lanthionine and homolanthione could be increased because their clearance is reduced, therefore representing new uremic retention products. 

However, since it is known that lanthionine is produced by intestinal bacteria in the synthesis of lanthibiotics [19], and it is known that the microbiota of uremic patients is severely altered [20,21], it is also possible that the observed increase in blood lanthionine could be linked to increased intestinal production and subsequent absorption [22].

## 4. Toxic Effects of Lanthionine

### 4.1. Lanthionine Hampers H_2_S Release In Vitro

**Figure 3:**
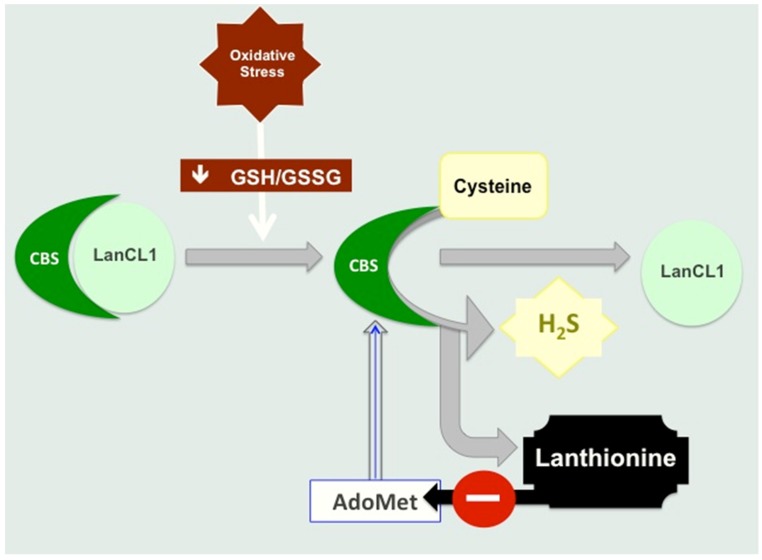
Proposed mechanism of inhibition of CBS by lanthionine. It has been shown that lanthionine synthetase C-like protein component 1 (LanCL1) directly binds and inhibits CBS [25]. LanCL1 is a sensor of oxidative stress. In particular, while LanCL1 is a negative regulator of CBS, oxidative stress down-regulates this binding and increases CBS activity. CBS and CSE catalyze cysteine biosynthesis from homocysteine (transsulfuration pathway); cysteine in turn is a key substrate for glutathione (GSH) biosynthesis. Alternatively, both CBS and CSE, independently, may commit cysteine and/or homocysteine sulfur metabolism to H_2_S biosynthesis. Regulation of the activity of CBS, a heme sensor protein, is very complex and multifaceted. Under ER stress conditions, CSE is induced and the CBS-inhibitor carbon monoxide (a product of heme oxygenase-1) is up-regulated. As a result, CSE preferentially utilizes cysteine, thus yielding H_2_S [9].

In addition, lanthionine is able to inhibit H_2_S formation in a hepatocarcinoma cell line (HepG2) cell model [10]. HepG2 expresses all the enzymatic machinery for sulfur amino acid metabolism, also including the enzymes of the transsulfuration pathway [17,23]. Therefore, HepG2 appears to be one of the most appropriate cell systems to study H_2_S production and the regulatory properties of the major enzymes involved, CBS and CSE. HepG2 cells were incubated with various substrates and cofactors for H_2_S production (homocysteine, cysteine, AdoMet, etc.) with or without lanthionine, at concentrations comparable to those measured in the uremic serum, utilizing the agar trap assay system. This method utilizes a modified zinc acetate procedure, in which an agar layer, poured on the top surface of a cell flask, is impregnated with zinc acetate during solidification. Cells were seeded and incubated overnight, in the presence of the substrate (Cys, Hcy) vitamin B6, AdoMet (see Figure 3), dl-lanthionine (at a concentration comparable to that detected in the uremic serum). H_2_S, released during cell incubation was trapped in the agar layer, and quantified in situ upon the addition of *N*,*N*-dimethyl-p-phenylenediamine sulfate and FeCl_3_ to each flask. Absorbance was finally read at 670 nm. The concentration was calculated against a standard curve obtained with a NaHS solution, as an H_2_S donor, attained under the above-mentioned conditions [10].

**Figure 4:**
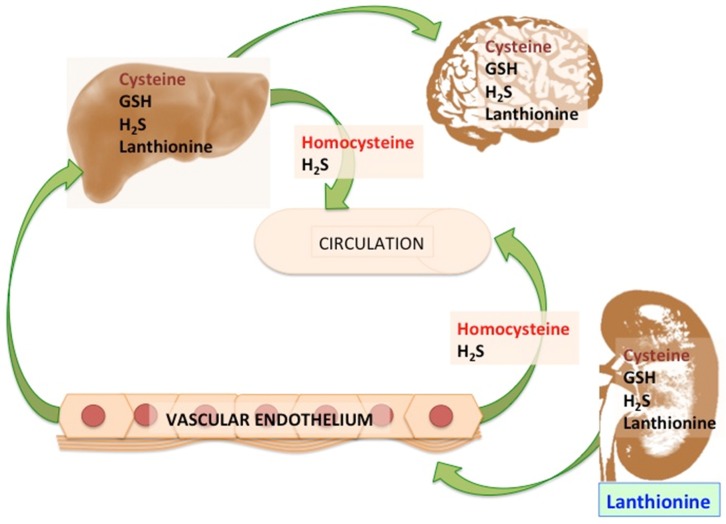
A tissue metabolic crosstalk hypothesis. CBS and CSE are reported to be differentially expressed in organs and tissues, thus allowing transsulfuration to be carried out preferentially, but not exclusively, in the liver, while H_2_S biosynthesis may be performed by a number of other cell types. In this respect, it has been shown that, in the liver, CBS protein levels are significantly lower than those of CSE, while both these enzymes are more abundant in the liver than at the kidney level [37]. So the hypothesis of a metabolic crosstalk depends on the reported non-ubiquitous expression of either CBS or CSE in various organs, leading to the suggestion that the transsulfuration pathway is incomplete in some tissues, where only CBS or CSE are prevalently expressed. Cysteine, the main product of transsulfuration may be mainly produced where transsulfuration is fully active, while its utilization occurs at many other levels even where this pathway cannot take place (e.g., RBC). Cysteine is a building block for glutathione biosynthesis, or it is the major substrate for H_2_S, carried out by both CBS and CSE independently, even in the tissues where only CSE is significantly expressed, yielding lanthionine as the main product. Evidence supports that lanthionine is a retention product during chronic renal failure, despite low levels of H_2_S being detected in these patients [10]. On the other hand, homocysteine, an obligate metabolic intermediate in the conversion of methionine into cysteine and a major demethylated product of transmethylation reactions, can be further metabolized by both transsulfuration and H_2_S biosynthetic reactions. One of the main side products of the latter reaction, which can be carried out by both CBS and CSE independently, is homolanthionine (see also Figure 1 and Figure 2). It should be remarked, however, that, in contrast with previous observations, the transsulfuration pathway has been detected using brain cells and slices, thus demonstrating its contribution to the glutathione-dependent redox-buffering capacity [38]. It has also been shown that the endothelium may contribute, as the liver, to the homocysteine metabolic process in which the transsulfuration enzymes, CBS and CSE, are secreted by microvascular endothelial cells and hepatocytes, circulate as members of the plasma proteome, and may actively produce hydrogen sulfide from homocysteine in blood [39]. The latter pathway is possibly impaired in uremia due to high lanthionine circulating levels. The illustrated scheme may become more complex as new evidence is added which allows us to clarify the quantitative contribution of each tissue/organ to the disposal of homocysteine and the formation of cysteine or H_2_S by trassulfuration enzymes. Black letters refer to products, red letters refer to substrates; lanthionine is susceptible to quantitative urinary excretion (blue); cysteine is both a transsulfuration product and a substrate for H_2_S biosynthesis (purple red).

Under these conditions, when lanthionine was present at concentrations in the range of what had been measured in vivo in hemodialysis patients, an inhibition of H_2_S release was found which accounted for the extra H_2_S release induced by AdoMet [10]. It then appears that the mechanism of the inhibition of H_2_S release, upon lanthionine cell treatment, may be mainly dependent on the ability of this compound to interfere with the allosteric activation of *S*-adenosylmethionine (AdoMet; SAM) on the enzyme CBS [24], one of the main enzymes involved in H_2_S biosynthesis in the liver (Figure 3). It should be pointed out, in this respect, that various organs and tissues may behave differently in their ability to use trassulfuration enzymes CBS and CSE to perform the complete transsulfuration pathway or, by using either one of the two enzymes, to synthesize H_2_S. A complementary crosstalk among various organs, differently regulated in their ability to use Cys (or metabolize Hcy), can be then hypothesized, in which cysteine produced in organs which are able to carry out the complete transsulfuration pathway may provide cysteine for GSH biosynthesis also for tissues which do not express both CBS and CSE (Figure 4).

It has also been reported that S-Glutathionylation increases CBS activity in response to oxidative stress [26].

Lanthionine is likely to interfere with AdoMet action upon CBS, since in our conditions, in the absence of the extra addition of this sulfonium compound, lanthionine inhibition of H_2_S release was abolished [10]. High circulating lanthionine in uremia could explain the H_2_S reduction and contribute at least in part to the degree of hyperhomocysteinemia in uremia, thus providing for a mechanism in the pathophysiology of this derangement.

### 4.2. Lanthionine Synthetase C-Like Protein (LanCL)

The eukaryotic lanthionine synthetase component C (LanC)-like proteins are homologues of prokaryotic LanC, which is a cyclase involved in the biosynthesis of lantibiotics in bacteria (“lanthionine-containing peptide antibiotics” [27]). The human genome encodes three lanthionine synthetase C-like proteins (LanCL), LanCL1, 2, and 3, the functions of which are largely unknown. LanCL1 was first isolated from human erythrocyte membranes [28]. It has been recognized as a prominent glutathione binding protein expressed in the mammalian central nervous system [29], and it inhibits CBS [25]. Increased levels of lanthionine ketimine ester, an analog of the natural sulfur amino acid metabolite lanthionine ketimine, have been found effective in a mouse model of multiple sclerosis [30]. LanCL1 is also a ligand for lanthionine ketimine, [12,31]. Lanthionine ketimine acts on the mammalian target of rapamycin (mTOR) pathway to stimulate autophagy in both neurotypic and glial cell cultures [32]. Human LanCL2 has been suggested to have a role in adriamycin sensitizing and the abscisic acid signaling pathway [33,34,35], and to be a novel regulator of Akt activity and cell survival [36]. LanCL1 and 2 have similar expression patterns, with strong expression in the brain and testis, and weak but ubiquitous expression in other tissues.

### 4.3. Lanthionine Concentrations as a Prospective Uremic Toxin

The molecular mass of lanthionine is 208.23; its concentration in the circulation in hemodialysis patients was detected in the low micromolar range (0.33 ± 0.03 μmol/L) and was almost undetectable in our non-uremic control population [10]. Conversely, indoxyl sulfate was detected at concentrations from three to four orders of magnitude higher, relevant to the free form and the protein-bound form, respectively [1]. Nevertheless, lanthionine concentrations detected in vivo in hemodialysis patients were effective to induce significant impairment of H_2_S release in vitro, in HepG2 cells [10].

The partitioning between free and protein-bound lanthionine in uremia has not been studied in great detail. We measured lanthionine and other sulfur amino acid metabolites, using LC-MS/MS, after sample deproteinization, which could have caused some loss of protein-bound lanthionine [10]. It is worth noting, in this respect that, firstly, lanthionine should not be expected to tightly bind to proteins, because of its low concentration and relatively high solubility. Secondly, it does not contain any thiol group, which could form covalent disulfides with human serum albumin. The latter is indeed the way thiol amino acids, such as Cys and Hcy, are generally transported in circulation, almost completely in the form of protein adducts (disulfides) with serum albumin. In addition, lanthionine was shown to be significantly removed by various types of hemodialysis techniques, thus confirming that the free form of lanthionine accounts for at least half of its concentration in circulation [10].

## 5. Conclusions

Results are consistent with the interpretation that lanthionine, in uremia, interferes with the mechanism of CBS activation by AdoMet. HepG2 cells stimulated with H_2_S precursors and cofactors (homocysteine, cysteine, AdoMet and vitamin B_6_) produced H_2_S and the amount increased with the duration of the incubation; lanthionine pre-treatment induced a significant decrease in the amount of H_2_S produced. The effect was comparable to that exerted by the transsulfuration inhibitor dl-propargyl glycine. Maximum H_2_S release was observed when cells were co-stimulated with AdoMet; in fact, when AdoMet was absent in the medium, the effect of lanthionine was abolished. Therefore, it is possible to hypothesize that lanthionine may interfere with AdoMet binding by increasing the stability of the LanCL1-CBS complex, thus increasing the inhibitory function of LanCL1 on CBS, leading to hyperhomocysteinemia (Figure 3 and Figure 4). It has been in fact suggested that hyperhomocysteinemia in uremia may be due to inhibition, by an unknown uremic toxin, of homocysteine-metabolizing enzymes, which is supported by the finding of reduced *CSE* transcription in mononuclear cells in vivo in uremic patients [15]. It can be hypothesized that the increase in blood levels of this compound found in uremic patients could contribute at least in part to the degree of hyperhomocysteinemia in uremia, thus providing a mechanism in the pathophysiology of this derangement, which is still not completely understood.
